# PET-Tool: a software suite for comprehensive processing and managing of Paired-End diTag (PET) sequence data

**DOI:** 10.1186/1471-2105-7-390

**Published:** 2006-08-25

**Authors:** Kuo Ping Chiu, Chee-Hong Wong, Qiongyu Chen, Pramila Ariyaratne, Hong Sain Ooi, Chia-Lin Wei, Wing-Kin Ken Sung, Yijun Ruan

**Affiliations:** 1 Information and Mathematical Sciences Group, Genome Institute of Singapore, 60 Biopolis Street, Genome #02-01, 138672, Singapore; 2 Bioinformatics Institute, 30 Biopolis Street, Matrix #08-01, 138671, Singapore; 3 Department of Computer Science, National University of Singapore, 3 Science Drive 2, 117543, Singapore; 4 Genome Technology and Biology Group, Genome Institute of Singapore, 60 Biopolis Street, Genome #02-01, 138672, Singapore

## Abstract

**Background:**

We recently developed the Paired End diTag (PET) strategy for efficient characterization of mammalian transcriptomes and genomes. The paired end nature of short PET sequences derived from long DNA fragments raised a new set of bioinformatics challenges, including how to extract PETs from raw sequence reads, and correctly yet efficiently map PETs to reference genome sequences. To accommodate and streamline data analysis of the large volume PET sequences generated from each PET experiment, an automated PET data process pipeline is desirable.

**Results:**

We designed an integrated computation program package, PET-Tool, to automatically process PET sequences and map them to the genome sequences. The Tool was implemented as a web-based application composed of four modules: the Extractor module for PET extraction; the Examiner module for analytic evaluation of PET sequence quality; the Mapper module for locating PET sequences in the genome sequences; and the ProjectManager module for data organization. The performance of PET-Tool was evaluated through the analyses of 2.7 million PET sequences. It was demonstrated that PET-Tool is accurate and efficient in extracting PET sequences and removing artifacts from large volume dataset. Using optimized mapping criteria, over 70% of quality PET sequences were mapped specifically to the genome sequences. With a 2.4 GHz LINUX machine, it takes approximately six hours to process one million PETs from extraction to mapping.

**Conclusion:**

The speed, accuracy, and comprehensiveness have proved that PET-Tool is an important and useful component in PET experiments, and can be extended to accommodate other related analyses of paired-end sequences. The Tool also provides user-friendly functions for data quality check and system for multi-layer data management.

## Background

Tag-based sequencing strategies such as Serial Analysis of Gene Expression (SAGE) are efficient for analyzing DNA fragments in transcriptome characterization and genome annotation studies [1-3]. However, the information content in each SAGE tag based on an anchored restriction enzyme recognition site within the DNA segment is limited, and the mapping of SAGE tags to genome sequences for transcript identification can be ambiguous. Despite the recent improvements in tagging 5' terminal signatures of cDNA [4,5] to determine transcription start sites (TSS), the most significant advance in this field is the simultaneous tagging of 5' and 3' terminal signatures of DNA fragments subjected to study. In this effort, we first developed an intermediate approach that precisely extracts separate 5' and 3' terminal tags from cDNA fragments for sequencing [6]. With this new capability, we proceeded to design and develop a cloning strategy, called Gene Identification Signature (GIS) analysis, which covalently links the 5' and 3' signatures of each full-length transcript into a Paired-End diTag (PET) structure [7]. In a GIS-PET experiment, most of the PETs are 36bp in length (18bp for the 5' signature tag and 18bp for the 3' signature tag); and multiple PETs can be concatenated together to form longer stretches of DNA fragments for efficient high-throughput sequencing. An average sequencing read (700–800bp) of a GIS-PET library clone can reveal 10–15 PET units, which is equivalent to 30 conventional cDNA sequencing reads for 15 cDNA clones analyzed from both ends. The PET sequences can then be accurately mapped to the reference genome sequences and precisely demarcate the boundaries of transcription units in the genome landscape. With this combined efficiency and accuracy of GIS-PET, a mammalian transcriptome can be thoroughly analyzed using hundreds of thousands high quality transcript sequences by a modest sequencing effort as further demonstrated in the comprehensive characterization of mouse transcriptomes [8]. The PET-based DNA analysis strategy has also been applied to characterize genomic DNA fragments generated by chromatin immunoprecipitation (ChIP) enriched for specific binding targets by given DNA-binding proteins, and whole genome ChIP-PET data has provided global maps of transcription factor binding sites for p53 in the human genome [9] and Oct4 and Nanog in the mouse genome [10]. PET-based DNA analyses (GIS-PET and ChIP-PET) promise to play a significant role in the post-genome efforts to identify all functional elements in the human genome [11], and there is no inherent limit for the PET-based approach to be applied to other DNA analyses, such as analyses of epigenetic elements.

To fully appreciate the potential of PET-based sequencing analyses, we have to develop sophisticated informatics capabilities to manage the large volume of specific PET sequences generated from each of the GIS-PET and ChIP-PET experiments. There is a battery of new bioinformatics challenges around how to accurately identify and extract PET sequences embedded in raw sequence reads, how to specifically and efficiently map the paired 5' and 3' signatures of PET sequences in complex genomes such as the human and mouse genome sequences; and how to be user-friendly in managing the immense amount of data generated from GIS-PET and ChIP-PET experiments for effective data mining and analysis. Based on the paired end nature of PET sequences generated from GIS-PET and ChIP-PET experiments, the issues are far more complicated than those related to SAGE-like mono-tags and therefore can not be handled by available software packages previously developed for SAGE analysis [12-15].

To accommodate and process PET sequence data, we developed a complete software suite called PET-Tool that is designed to provide complete solutions starting from extracting PET sequences from raw sequencing reads, to mapping the PET sequences to the reference genomes. Here in this study, we describe the architecture design, technical details of implementation, utility, and robustness of PET-Tool by analyzing four datasets generated from two GIS-PET libraries and two ChIP-PET libraries.

### The architecture of PET-Tool

**Figure 1:**
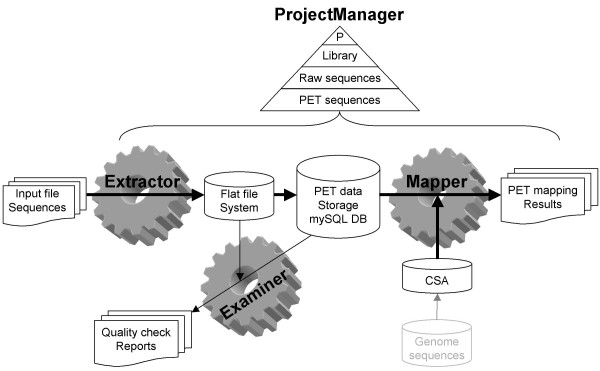
**A schematic view of architecture design for PET-Tool. **PET-Tool has four functional modules, Extractor, Examiner, Mapper, and ProjectManager. Extractor uploads sequence files and dissects the PET sequences from raw sequences. Examiner provides analytical functions for users to evaluate the extraction results and PET sequence quality. Mapper searches the genome database for the mapping locations of the PET sequences. ProjectManager is a hierarchical information management system. 'P' stands for "project". 'CSA' stands for "compressed suffix array", which in this instance is derived from the human genome assembly hg17.

PET-Tool is designed to provide complete solutions for processing and managing the PET data generated from GIS-PET and ChIP-PET experiments. In these experiments, either full-length cDNA or genomic DNA enriched by ChIP are converted into PET structures that are further concatenated and cloned into plasmid vector for sequencing analysis [7,9]. The core functions of the Tool are to extract PET sequences from raw DNA sequence reads and map the PET sequences to the genome sequences. In addition, we want the Tool to be able to manage large volume of PET data generated from each PET experiment and provide user-friendly analytic functions to evaluate the quality of each PET dataset. The design of PET Tool is comprised of four modules: Extractor, Examiner, Mapper, and ProjectManager (Figure 1). In the PET-Tool system, Extractor uploads raw sequence files and de-convolutes the PET sequence units embedded in each raw sequence read to generate PET sequences, which are stored in a relational database. Examiner provides an analytical capability for users to examine and validate the PET extraction results. It provides the basic statistics of PETs in each project, library, and plate of sequences. It also presents graphic dissection for each of the input sequence reads and highlights the sequence sections with various color codes to help users to distinguish vector flanking regions, spacer sequences between PET units, and the PET sequences themselves. This ability allows users to identify any potential irregularities in the sequence, and adjust extraction parameters. The Mapper module is to map the quality PET sequences to the corresponding genome sequences. For efficient mapping of large volumes of PET sequences, we used a newly developed alignment approach that was based on compressed suffix array (CSA), in which the entire genome sequence assembly was indexed as a reference database, and the 5' and 3' signatures of a PET sequence were matched to the genomic index [unpublished results]. The ProjectManager module organizes the data and analysis results in a hierarchical order, in which multiple projects can be managed at various levels of organisms, libraries, raw DNA sequences (in plate and single well format), PETs, and the attributes of each PET.

### Implementation

PET-Tool is implemented for both UNIX and LINUX. The web-based user interface is implemented in Perl/CGI and hosted by Apache web server. The interface of the Tool can be accessed by any web-browser that supports the current web standards.

Data storage is facilitated by a combination of flat file system and mySQL based Relational Database Management System (RDBMS). The mySQL database was used for efficient and fast PET data storage, tracking, retrieving, and interfacing with back-end programs through Perl:DBI module. We also applied mySQL to host various statistical data and mapping results. Flat files were used for storage of uploaded sequence data, with the positional indices of all sequences stored in mySQL database for quick sequence retrieval. Back-end programs were implemented in Perl and C languages. Compressed Suffix Array (CSA) programs were implemented in C language for high efficiency and robust performance of advanced data structures. Programs for PET sequence extraction, statistic computation, data retrieval/storage, web-interaction and other non-intensive tasks were implemented in Perl. Minimum hardware requirements include Pentium III processor, CPU of 500 MHz, 256 Mega byte RAM, and 20 Giga-byte hard disk drive. A regular 500 MHz machine would take about two days to process a library of one million PETs. If a computer was equipped with 2.4 GHz processor, the same job could be done in a few hours.

## Results and discussion

The current settings of PET-Tool can handle GIS-PET for transcriptome analysis and ChIP-PET data for whole genome localization of transcription factor binding sites. We have successfully applied PET-Tool to more than 45 GIS-PET and ChIP-PET libraries. To demonstrate the data processing workflow, and the functionalities and performance of PET-Tool, we analyzed two GIS-PET libraries and two ChIP-PET libraries in this study.

### Datasets used for analysis in this study

The two GIS-PET libraries (SHC12 and SHC13) used in this study were derived from human cancer cells MCF7 and HCT116, respectively; and the two ChIP-PET libraries (SHC16 and SHC19) were from chromatin immunoprecipitated DNA enriched for STAT1 binding sites in human HeLa cells with and without interferon-γ treatment, respectively. For each of the PET libraries, more than 50,000 plasmid clones were sequenced from one direction. In total, over 300,000 high quality sequence reads were generated for these 4 libraries (Table 1).

### PET-Tool procedure to process the PET library sequences

**Figure 2:**
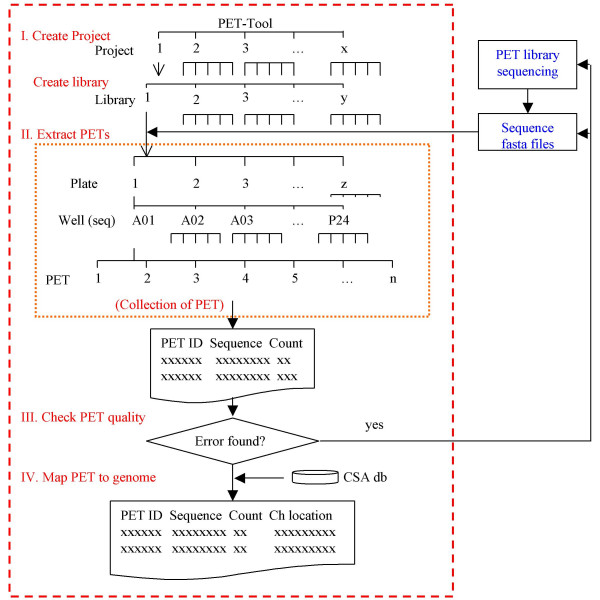
**Process flow of PET data Analysis in PET-Tool. **Experimental information for each PET library was entered into the system through the ProjectManager functions. High quality DNA sequences in the FASTA files of PET libraries were uploaded through Extractor. Extracted PETs and all related information were stored in a mySQL database. Virtually, PETs were organized in a hierarchical order from project to library, plate, well, and individual PTE. Each unique PET was assigned a unique ID and accorded with copy number (count). The quality of PET extraction was evaluated using Examiner. Errors that occurred in any steps in PET extraction or PET library construction and sequencing could be identified for correction. After PET extraction was validated, PET sequences were mapped to the genome sequences, and the mapping coordinates for each of the PETs were reported in table format.

The use of PET-Tool starts from the ProjectManager module, from which new project and library IDs were assigned for each new sequence dataset. In the PET-Tool system, a project can host multiple libraries, and a library can contain from a few to hundreds of thousands of sequences. The two GIS-PET libraries presented in this study were assigned library IDs SHC012 and SCH013 under the project name "Human Transcriptome", while the two ChIP-PET libraries were assigned as SCH016 and SCH019 under the project name "Human TFBS" (transcription factor binding sites). Once the entry for a library is created, the library sequence file is uploaded and the PET sequences are extracted from raw DNA sequences via the **Extractor **module. The quality of PET sequences can be examined using the Examiner module, and high quality PET sequences can be mapped to the corresponding genome sequences using the Mapper module (Figure 2).

### PET sequences generated from GIS-PET and ChIP-PET libraries

**Figure 3:**
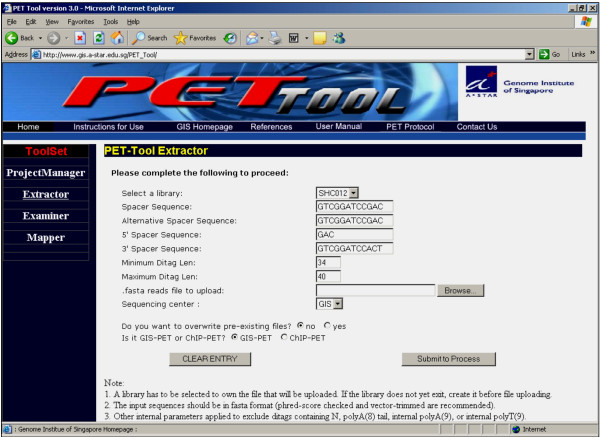
**PET extraction using the Extractor module. **The process of PET extraction is initiated through the opening of 'Extractor' listed under the 'ToolSet'. Once a library is selected, library-specific parameters related to PET extraction will show up, such as spacer sequences and minimum/maximum PET length previously entered during library creation. Once the extraction parameters are confirmed or modified by the users, the DNA sequence file in FASTA format is browsed and uploaded. The 'sequencing center' accommodates different naming conventions for sequence IDs (used in input files) generated by different sequencing centers. The selection of a given naming convention method from the 'sequencing center' is needed for the system to properly parse individual sequences in groups of specific wells and plates for particular libraries. The user also needs to specify if the library is a GIS-PET or ChIP-PET library.

Based on library construction methods, specific parameters for PET extraction such as the nucleotide sequences for 5' and 3'spacers flanking plasmid vector, the internal spacers between two PET units, and the expected PET length, were entered into the system in the front page of the ProjectManager during library creation and can be modified through the Extractor module during sequence file uploading. For the two GIS-PET libraries, 'GAC' was the 5' spacer, 'GTCGGATCCGAC' was the internal spacer and 'GTCGGATCCACT' was the 3' spacer. For the two ChIP-PET libraries, the spacers used for library SHC019 were the same as for the GIS-PET libraries, while SHC016 used a different set of spacer sequences, in which the 5' spacer was 'AC', the internal spacer 'GTCGAC', and the 3' spacer 'GTCGATC'. Once the extraction parameters were defined, each of the library sequence files in FASTA format was uploaded (Figure 3) and stored in the flat file system. In the Extractor module, DNA sequences were read through, all sequence sections resided in between two spacer sequences were distinguished, and potential PET sequences were extracted. From the 128,295 raw sequence reads of the two GIS-PET libraries, 1,105,762 PETs were generated; while from the 172,300 raw sequences of the two ChIP-PET libraries, 1,622,083 PETs were generated. The overall efficiency of PET production is 8.6 PETs per sequence read for the two GIS-PET libraries and 9.4 PETs per sequence read for the two ChIP-PET libraries.

The spacer-defined raw PETs were then subjected to serial steps of filtering to exclude incorrect PETs due to imperfect molecular reactions during the molecular cloning process. It is known that the TypeIIs restriction enzymatic cleavage, DNA end polishing, and ligation reactions have a certain level of slippage, and the combination of these reactions would contribute to deviation of actual PET lengths from the predicted PET lengths by one to several nucleotides [7]. Hence, we have set an empirical range (34–40bp) around the expected size (36bp) for true ditags. Other ditags that were either shorter than 34 bp or longer than 40 bp were considered experimental artifacts, and therefore were removed from further analysis. PET sequences with low complexity (homopolymer stretches of more than 8 consecutive same nucleotides such as As or Ts, etc) were also removed because these PETs lack sufficient specificity for mapping to reference genome sequences. As an indication of PET orientation, we kept an "AA" residue of the cDNA polyA tail in the PET sequences at 3' end in GIS-PET libraries. Therefore, if any GIS-PET ditags did not contain the AA tail at the 3' end, these questionable PETs were also removed. After these layers of filtering, 864,964 high quality PETs were collected for the two GIS-PET libraries and 1,489,412 high quality PETs for the ChIP-PET libraries. Redundant PETs were collapsed into unique PETs. The copy numbers for each of the unique PETs reflect the abundance level of the PET in a given library. In total, 135,757 unique PETs were collected for SCH012, 145,138 for SCH013, 640,844 for SCH016, and 582,253 for SCH019 (Table 1).

### Evaluation of PET quality using examiner

**Figure 4:**
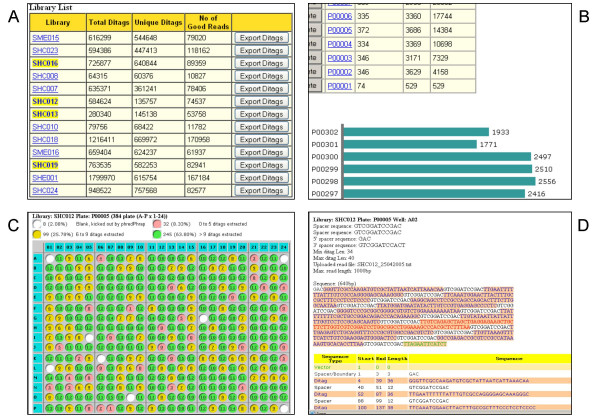
**Functions of the Examiner module. **PET extraction results can be viewed at various levels (library, plate, well, and nucleotide sequence). **A. **The library view of PET extraction result. The 4 libraries analyzed in this study were highlighted. The numbers of total PETs, unique PETs, and high quality sequence reads used for PET extraction are shown. **B. **The plate view of PET extraction results. Individual plates (plate ID) and the number of quality sequences, total PETs and cumulative PETs are shown in table format as well as graphic bar display. **C. **The 384-well view of PET extraction results. The digit in each well stands for the number of PETs generated from the sequence in that well. Each well is also color coded for 4 different categories based on the number of PETs produced. The 4 categories are summarized at the top panel of the table. **D. **Individual sequence view of PET extraction results. A sequence was dissected into spacer sequences and the putative PET sequences in between two adjacent spacers. The spacer sequences are in black and with plain background, and the sequences in between spacers are highlighted in orange color. The good PET sequences are in blue, and the bad sequences in red. The sequence segments are further tabulated with detailed information regarding the position and the length of each segment.

When the automated process of PET extraction was complete, the PET sequences with related information were organized in mySQL tables. The PET extraction results can be viewed using Examiner. To add analytical functions for evaluating the library sequencing quality, any particular sequencing plate and individual sequencing wells can be viewed for PET extraction results. Furthermore, in the Examiner module, the pattern of PETs and spacers embedded in the original sequence reads can be displayed with color codes at the nucleotide level (examples of screenshots are shown in Figure 4). This level of detail in validating the quality of PET library sequences and the accuracy of PET extraction results provide a great convenience for users.

### Comparison of PET sequences derived from GIS-PET and ChIP-PET libraries

Although the methods used to generate GIS-PET and ChIP-PET were similar, the starting DNA materials were rather different. GIS-PETs were derived from cDNA, while ChIP-PETs were derived from ChIP enriched genomic DNA fragments. It appears that the quality of GIS-PETs is lower than that of ChIP-PETs. About 22% of GIS-PET sequences as opposed to 8.2% of ChIP-PET sequences were rejected after quality filtering. There are several reasons contributing to higher error rates for GIS-PETs. One of the major differences between GIS-PET and the ChIP-PET was the inclusion of AA-tail as a 3' directional indicator at the end of 3' signature for each GIS-PET sequence. We observed that 30% of the rejected GIS-PETs lacked the appropriate AA-tail. We also observed that the AT content in GIS-PETs was significantly polarized, at 31% for the 5' tag region and 61% for the 3' tag region. This observation is in consistent with our knowledge that in transcripts or cDNAs, 5' UTR (un-translated region) is GC rich and 3' UTR is AT rich [16]. In contrast, the 3'-prone polarization of AT content was not observed in ChIP-PET sequences because the ChIP DNA fragments were generated by randomly shearing of genomic DNA.

### Mapping of PET sequences to reference genome sequences

**Figure 5:**
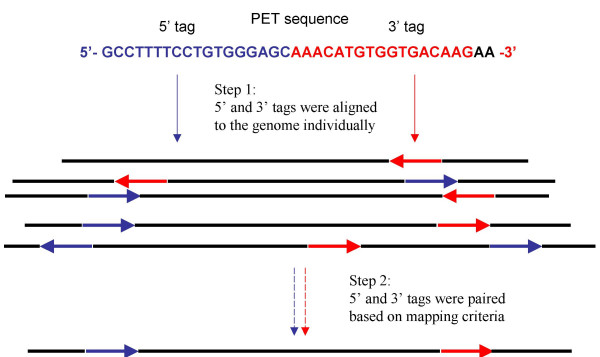
**Mapping of PETs to the genome. **The 5' and 3' tags of a PET were split and separately mapped to the human genome sequences. Due to the short length of tags and the complexity of the human genome, some of the tags could be mapped non-specifically to multiple locations in the genome. The 5' and 3' tags derived from the same PET were mated based on the criteria that the paired 5' and 3' tags had to be in the correct orientation and order (5'→3'), on the same chromosome, and within the defined appropriate distance.

**Figure 6:**
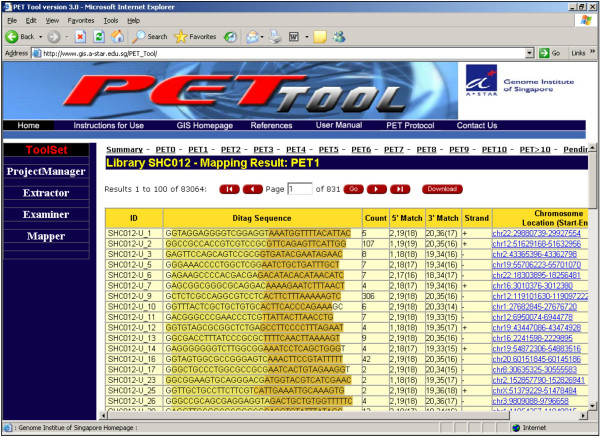
**Mapping report of a PET library. **The PET mapping results of a library were tabulated and reported. The table included the ID number for each of the unique PETs, PET sequences, PET counts, alignment specificities for 5' and 3' tags, PET mapping orientations (DNA strand, + or -), and PET genomic coordinates.

Once the PET extraction results were confirmed, the PET sequences were ready for mapping to the human genome sequences. Using the Mapper module, each of the PET sequences was split into a 5' tag and a 3' tag, and the tags were independently searched for matches in of the human genome assembly (hg17). When mapped correctly to the genome sequences, a PET sequence from nucleotide position 1–18 should be aligned to the 5' boundary and 19-last to the 3' boundary of the corresponding DNA segment on a chromosomal locus. However, considering that both reverse transcriptase-derived non-templated nucleotide incorporation and TypeIIs restriction enzyme slippage could lead to ambiguities at the two ends of individual tags in a PET structure [6], we mandated a minimum 16-nucleotide contiguous match for the 5' (from nucleotide position 1 to 19) and 3' (from 18 to the last) tags of PET to accommodate most possible variations. The mapped tags were then mated based on the criteria that the paired 5' and 3' signatures of a PET sequence must be on the same chromosome, in the correct order and orientation (5'→3'), and within appropriate genomic distance. Human genes encoded in chromosomes contain intron/exon structures, and many of them could span large distance of the genome for (hundreds of thousands of base pairs). To accommodate most genes but set a limit to reduce non-specific matches, we decided on an arbitrary cutoff of one million base pairs between 5' and 3' tags of GIS-PETs. For the ChIP-PET sequences, since the ChIP enriched DNA fragments were ranged from less than 100 base pairs to the up-limit of 4000 base pairs as estimated by DNA electrophoresis on agarose gel, the cutoff for ChIP-PET mapping span was set at 6000 bp. This criterion for pairing within a defined size limit greatly increased the mapping specificity of PET to genome sequences, and non-specific mapping of individual tags were most likely not fit in such mating requirement (Figure 5). The final mapping and pairing results were reported in a mySQL table format that included the PET nucleotide sequences, the count (copy number) for each of the PET sequences, nucleotide positions of 5' and 3' matches, and genome coordinates to which the PET sequences mapped (Figure 6). These data provide detailed information, not only where PET sequences mapped, but also the frequency for each of PETs occurred in each library.

As presented in the four PET libraries in this study, more than 70% of PETs were mappable. For example, of the total unique PETs generated for Library SCH012, 102,660 PETs (75.6%) were mapped accurately to the human genome assembly hg17 (Table 2). The majority of the mapped PETs (83,089/102,660; 80%) mapped to single location in the genome. The rest of the PETs mapped to two or more locations in the genome, which may reflect the mapping of PETs to duplicated gene segments and repetitive elements in the genome. It is noteworthy that more than 92% of the mapped ChIP-PETs found a unique position in the genome. As we reported in previous studies [7,9], most PET sequences were accurate in demarcating the corresponding DNA elements in the genome. For example, over 98% of the GIS-PETs mapped accurately to 5' and 3' boundaries of known gene transcripts.

## Conclusion

We have developed a comprehensive computation program package, PET-Tool, to accommodate demands for automated processing of large volume of PET sequences generated by PET-based experiments. We demonstrated the utility of PET-Tool by analyzing four PET libraries and more than 2.7 millions PET sequences, and proved that PET-Tool can accurately and efficiently dissect PET concatemer sequences, extract, organize PET sequences in a relational database for convenient evaluation of sequence quality and overall experimental integrity, and specifically map the PET sequences to the corresponding reference genome sequences.

## Availability and requirements

Project name: PET-Tool; Project home page:  Operating system(s): UNIX and LINUX; Programming language: Perl and C languages.

PET-Tool is free for non-commercial use. The complete package of PET-Tool is available in DVD format to be sent upon request, and downloadable from the PET-Tool home page. For users who would like to understand more of the PET methodology, a detailed experimental protocol and a user manual are also available at the PET-Tool website.

## Abbreviations

SAGE: Serial Analysis of Gene Expression

ChIP: chromatin immunoprecipitation

PET: Paired-End diTag

GIS: Gene Identification Signature

CSA: Compressed Suffix Array

TSS: Transcription Start Site

PAS: Poly-Adenylation Site

## Authors' contributions

CLW and YR conceptualized the framework of PET-Tool. KPC was overall responsible for detailed design and implementation of the programs, and involved in coding some of the Perl programs, as well as data curation. CHW was responsible for mySQL database design and coding of related Perl programs. WKS and QC developed the CSA programs used for PET mapping. PA and HSO helped to modify the programs. CLW provided user inputs and evaluated the prototype. WKS provided computational advice to improve the overall performance. KPC and YR wrote the paper.

## Figures and Tables

**Table 1:** Statistics of PET characteristics

**Library Type**	**GIS-PET**			**ChIP-PET**		
**Library ID**	**SHC012**	**SHC013**	(combined)	**SHC016**	**SHC019**	(combined)
**Raw sequence reads**	74,537	53,758	128,295	89,359	82,941	172,300
**Spacer-defined PETs**	741,799	363,963	1,105,762	777,038	845,045	1,622,083
**PET/sequence read**	10	6.8	8.6	8.7	10.2	9.4
**Rejected poor PETs**	157,175	83,623	240,798	51,161	81,510	132,671
Rejection rate % *	21.1	22.3	22	6.6	9.7	8.2
**Total high-quality PETs**	584,624	280,340	864,964	725,877	763,535	1,489,412
**Total unique PETs**	135,757	145,138	280,895	640,844	582,253	1,223,097
Redundancy % **	76.8	48.2	62.5	11.7	23.7	17.7
**5' AT content (%)**	31.71	32.89	32.30	58.06	57.85	57.96
**3' AT content (%)**	61.49	62.18	61.84	57.98	57.57	57.78

**Breakdown of rejected PETs**	157,175	83,623	240,798	51,161	81,510	132,671
**Length < 34**	43,022	7,942		26,343	64,900	
	-27.40%	-9.50%		-51.10%	-79.60%	
**Length > 40**	17,377	7,967		24,794	16,581	
	-11.10%	-9.50%		-48.90%	-20.40%	
**Contain N**	16	3		24	29	
**No AA-tail at 3' end**	43,673	26,901	-30.00%	N. A.	N. A.	
	-27.80%	-32.20%				
**PolyA(9) in 3' tag**	41,983	20,264	-25%	N. A.	N. A.	
	-26.70%	-24.20%				
**PolyA(9) in 5' tag**	433	99		N. A.	N. A.	
**PolyT(9) in 5' tag**	116	118		N. A.	N. A.	
**PolyT(9) in 3' tag**	639	323		N. A.	N. A.	

* Rejection rate = "Rejected poor PETs"/"Spacer-defined PETs". ** Redundancy = (1-Total unique PETs/Total high quality PETs) × 100. The number states the percentage of PET tags that are redundant in the category.

**Table 2:** PET mapping to the genome

**Library Type**	**GIS-PET**		**ChIP-PET**	
**Library ID**	**SHC012**	**SHC013**	**SHC016**	**SHC019**
**Unique PETs**	135,757	145,138	640,844	582,253
**Un-mapped PET**	33,097	34,648	183,314	166,764
**(un-mapping rate %)**	24.4	23.4	28.6	28.6
**Mapped PET**	102,660	110,490	457,530	415,489
**(mapping rate %)**	75.6	76.1	71.4	71.4
**PETs mapped to one location (PET1)**	83,089	88,850	422,145	382,788
**(PET1 rate% to unique PETs)**	61.2	61.2	65.9	65.7
**(PET1 rate % to mapped PETs)**	80.9	80.4	92.3	92.1
**(PET1 tag total counts)**	372,937	174,443	473,224	516,453
(PET1 redundancy %)*	77.7	49.1	12.1	24.3
**PET1 mapped to known genes**	78,905	85,031	NA	NA
**(PET1 rate % to known genes)**	93.6	94.3	NA	NA

**PETs mapped to multiple locations (% to all mapped PETs)**				
**PET2 (%)**	7.67	8.16	1.87	1.94
**PET3 (%)**	2.33	2.37	0.68	0.71
**PET4 (%)**	1.2	1.26	0.41	0.44
**PET5 (%)**	0.64	0.72	0.29	0.28
**PET6 (%)**	0.43	0.48	0.23	0.2
**PET7 (%)**	0.31	0.31	0.17	0.17
**PET8 (%)**	0.18	0.18	0.15	0.15
**PET9 (%)**	0.16	0.15	0.11	0.12
**PET10 (%)**	0.11	0.11	0.09	0.09
**PET11+ (%)**	1.38	1.17	1.52	1.53

* PET1 Redundancy % = (1-Total unique PETs/Total high quality PETs) × 100. The number states the percentage of PET tags that are redundant in the category.
